# Duck Tembusu Virus Utilizes miR-221-3p Expression to Facilitate Viral Replication *via* Targeting of Suppressor of Cytokine Signaling 5

**DOI:** 10.3389/fmicb.2020.00596

**Published:** 2020-04-21

**Authors:** Min Cui, Shuling Chen, Shaqiu Zhang, Anchun Cheng, Yuhong Pan, Juan Huang, Zhiqiang Hu, Xingcui Zhang, Mingshu Wang, Dekang Zhu, Shun Chen, Mafeng Liu, Xinxin Zhao, Yin Wu, Qiao Yang, Yunya Liu, Ling Zhang, Yanling Yu, Zhongqiong Yin, Bo Jing, Mujeeb Ur Rehman, Bin Tian, Leichang Pan, Renyong Jia

**Affiliations:** ^1^Avian Diseases Research Center, College of Veterinary Medicine, Sichuan Agricultural University, Chengdu, China; ^2^Institute of Preventive Veterinary Medicine, Sichuan Agricultural University, Chengdu, China; ^3^Key Laboratory of Animal Disease and Human Health of Sichuan Province, College of Veterinary Medicine, Sichuan Agricultural University, Chengdu, China

**Keywords:** duck Tembusu virus, miR-221-3p, interferon beta, suppressor of cytokine signaling 5, immune response

## Abstract

Duck Tembusu virus (DTMUV), a member of *Flaviviridae* family, causes acute egg-drop syndrome in ducks. MicroRNAs (miRNAs) have been found to be involved in various biological processes, including tumor genesis, viral infection, and immune response. However, the functional effect of miRNAs on DTMUV replication remains largely unclear. This study aimed to elucidate the role of host microRNA-221-3p (miR−221-3p) in regulating DTMUV replication. Here, we indicated that the expression of miR−221-3p was significantly upregulated in duck embryo fibroblasts (DEFs) during DTMUV infection. Transfection of miR-221-3p mimic significantly reduced interferon (IFN) β production, whereas transfection of miR-221-3p inhibitor conversely significantly increased the expression of IFN-β in DTMUV-infected DEF. Moreover, we found that viral RNA copies, viral E protein expression level, and virus titer, which represent the replication and proliferation of virus, were all enhanced when transfecting the miR-221-3p mimic into DEF; reverse results were also observed by transfecting the miR-221-3p inhibitor. We also found that the expression of suppressor of cytokine signaling 5 (SOCS5) was downregulated in DEF infected with DTMUV. Besides, we further proved that SOCS5 is a target of miR-221-3p and that miR-221-3p could negatively modulate SOCS5 expression at both mRNA and protein levels. Finally, our results showed that overexpression of SOCS5 inhibited DTMUV replication and knockdown of SOCS5 enhanced DTMUV replication. Thus, our findings reveal a novel host evasion mechanism adopted by DTMUV *via* miR-221-3p, which may hew out novel strategies for designing miRNA-based vaccines and therapies.

## Introduction

Duck Tembusu virus (DTMUV) belongs to the genus *Flavivirus* of the *Flaviviridae* family. DTMUV was first detected in China in 2010, then quickly spread to most regions of China, causing serious economic losses in the duck industry (Jingliang et al., 2011; Pixi et al., 2011; Zhenzhen et al., 2011). In recent years, the infection of TMUV has also emerged in chickens, geese, and sparrows (Tao et al., 2012; Tang et al., 2013a; Shilong et al., 2014; Ti et al., 2015). More importantly, the potential case of human infection by TMUV was also reported in recent research, suggesting that the virus may pose a threat to public health (Tang et al., 2013b).

miRNAs, a highly conversed non-coding small RNAs of about 22nt in length, play key regulator roles in gene expression through transcription repression or mRNA destabilization *via* suppression of gene expression by binding to the complementary sequences in 3-untranslated region (UTR) of target mRNAs (Bartel, 2004; Lin and Hannon, 2004; Victor, 2004; Fabbri et al., 2007). It is now well documented that there is a correlation between miRNAs expression and viral infection (Ojha et al., 2016; Trobaugh and Klimstra, 2017; Hussein et al., 2019; Islam et al., 2019; Sartorius et al., 2019). In particular, recent studies further suggested that the expression profiles of host miRNAs were changed or viral miRNAs were generated during viral infection (Skalsky and Cullen, 2006; Kincaid and Sullivan, 2012; Castro et al., 2019). In turn, these host and viral miRNAs can also affect the process of virus infection *via* targeting viral genome or host genes (Sullivan and Ganem, 2005; Scaria et al., 2006; Ojha et al., 2016). For instance, a direct role of miR-34b enhancing avian leucosis virus (ALV-J) replication was revealed by repressing melanoma differentiation-associated gene 5 (MDA5) expression (Li et al., 2017). Further, increasing studies have also proved that miRNAs could also regulate flaviviral infection. As an illustration, the members of miR-34a family inhibited the replication of Dengue virus (DENV), West Nile virus (WNV), and Japanese encephalitis virus (JEV) *via* repression of Wnt signaling and activation of interferon (IFN) response (Smith et al., 2017). miR-281, let-7c, miR-484, and miR-744 modulated DENV-2 replication through targeting the DENV genome (Zhou et al., 2014; Escalera-Cueto et al., 2015; Castrillon-Betancur and Urcuqui-Inchima, 2017). miR-532-5p restricted WNV infection by targeting two host genes, including SEC14 and spectrin domain containing 1 (SESTD1) and transforming growth factor-b-activated kinase 1/MAP3K7 binding protein 3 (TAB3) (Slonchak et al., 2015). Taken together, the effects of miRNAs on these flaviviral infections (including JEV, DENV, and WNV) have already been extensively studied. However, the regulatory role of host miRNAs during the progress of DTMUV infection remains unknown.

Furthermore, there are growing evidences that showed that miRNAs regulate the infection process of various viruses by regulating the immune response of host organisms (Xiao and Rajewsky, 2009; Lu and Liston, 2010; Urcuqui-Inchima et al., 2017; Annie and Selena, 2018; Momen-Heravi and Bala, 2018). Several cellular miRNAs were utilized by viruses to evade host immunity. For example, miR-132 was upregulated in three herpesvirus infection, including herpes simplex virus type 1 (HSV-1), Kaposi’s sarcoma-associated herpesvirus (KSHV), and human cytomegalovirus (HCMV). The miR-132 negatively modulated IFN-stimulated genes (ISGs) production through suppressing the expression of the p300 transcriptional coactivator, which results in immune evasion of the three herpesvirus infection (Lagos et al., 2010). It is also reported that HSV-1 exploited miR-23a to avoid host immune response *via* targeting IFN regulatory factor 1 (IRF1), finally facilitating HSV-1 replication (Jing et al., 2014). During JEV infection, miR-301a inhibited antiviral IFN response and promoted immune evasion by targeting IRF-1 and cytokine signaling 5 (SOCS5) (Hazra et al., 2017). Similarly, miR-146a has been found to be involved in the immune evasion of DENV and JEV infection. It was confirmed that miR-146a prevented IFN response *via* targeting tumor necrosis factor receptor-associated protein 6 (TRAF6), thus promoting the infection of DENV and JEV (Wu et al., 2013; Sharma et al., 2015). Therefore, we aimed to uncover the complex relationships between miRNAs and host immune response during DTMUV infection.

In the previous study, we have identified the miRNA profiles in DTMUV-infected and uninfected duck embryo fibroblast (DEF), and we found that 48 cellular miRNAs were dysregulated, including miR-221-3p (Cui et al., 2018). Currently, miR-221-3p has been shown to be overexpressed in many tumor cells and has become a unique marker of the tumor (Xing Xiang et al., 2010; Chen et al., 2013; Kawaguchi et al., 2013; Xing Xing et al., 2014). Recent research studies have shown that miR-221 is also associated with viral infection (Xu et al., 2014; Du et al., 2018). For instance, miR-221 was found to repress HCV replication by targeting suppressor of cytokine signaling 1 (SOCS1) and suppressor of cytokine signaling 3 (SOCS3) (Xu et al., 2014). A recent study demonstrated that miR-221 negatively regulated innate immune response and promoted VSV and HSV-1 replication (Du et al., 2018). However, whether the upregulated miR-221-3p in DTMUV-infected DEF will affect the replication of DTMUV has not been reported until now.

In this study, we chose a significantly upregulated miR-221-3p to investigate its effect on IFN production and DTMUV replication. Our results showed that miR-221-3p mimic transfection inhibited the production of IFN-β, while it increased DTMUV E protein expression, the viral RNA copies, and virus titer. Then we further proved that miR-221-3p could downregulate SOCS5 expression *via* binding the 3′-UTR of SOCS5. We also found that DTMUV replication was enhanced through knockdown of SOCS5. Taken together, our results showed that miR-221-3p facilitated DTMUV replication *via* the downregulation of SOCS5. Thus, our findings unveil a novel host evasion mechanism adopted by DTMUV through miR-221-3p, which may open up a new avenue for therapeutic intervention.

## Materials and Methods

### Ethics Approval and Consent to Participate

The usage of duck embryos and ducks in this study was approved by the Animal Ethics Committee of Sichuan Agricultural University (approval no. XF2016-17) and followed the National Institutes of Health guidelines for the performance of animal experiments.

### Cell and Virus

DEFs were prepared from 9-day-old duck embryos and cultured in Dulbecco’s modified Eagle medium (DMEM) (12800017, Gibco, United States) supplemented with 10% fetal bovine serum (FBS) (16010159, Gibco, United States) at 37°C in 5% CO_2_ incubator. The DTMUV CQW strain (GenBank: KM233707.1) was obtained from the Key Laboratory of Animal Disease and Human Health of Sichuan Province. For viral infection, monolayer DEF cells were infected with the DTMUV CQW strain at a multiplicity of infection (MOI) of 1 in DMEM supplemented with 2% FBS.

### miRNA Target Prediction

The potential miRNA target genes were predicted by RNAhybrid and miRanda at default settings (Krek et al., 2005; Rajewsky, 2006).

### Transfection of miRNA Mimic and Inhibitor

miR-221-3p mimic, mimic negative control (mimic-NC), miR-221-3p inhibitor, and inhibitor negative control (inhibitor-NC) were purchased from RiboBio (Guangzhou, China). DEF cells were seeded into 6-well or 12-well plates and cultured in DMEM with 10% FBS at 37°C. When the DEF cells were grown to 70–80% confluence, the miRNA mimic (100 nmol), mimic-NC (100 nmol), inhibitor (200 nmol), and inhibitor-NC (200 nmol) were transfected into the cells using Lipofectamine 3000 (Invitrogen, United States) according to the manufacturer’s protocol. After 24 h of transfection, the DEF cells were infected with the DTMUV CQW strain. Then, the cells were collected for future analysis at 36 or 72 h after infection.

### Knockdown and Overexpression of Suppressor of Cytokine Signaling 5

Three shRNAs against SOCS5 were synthesized in Shanghai GenePharma Co, Ltd.; and pcDNA3.1(+)-SOCS5 plasmid was constructed. DEF cells were seed into 6-well plates and cultured at 37°C in 5% CO_2_ incubator. When the DEF cells were grown to 70–80% confluence, SOCS5 shRNAs, pcDNA3.1(+)-SOCS5, and their respective controls were transfected into DEF cells using Lipofectamine 3000 according to the manufacturer’s protocol. After 24 h of transfection, the DEF cells were infected with DTMUV, and the cells were collected for future analysis at the indicated time.

### RNA Isolation and Quantitative Real-Time PCR

Total RNA from treated DEF was isolated using RNAiso plus reagent (9109, TaKaRa, Japan) according to the manufacturer’s instruction. The concentration of total RNA was determined by NanoDrop 2000 (Thermo Fisher, United States). The primers used in qRT-PCR are listed in Table 1. For miRNA quantification, cDNA was prepared with stem-loop structured reverse primers using PrimeScript^TM^ RT Master Mix (RR036A, TaKaRa, Japan). For mRNA quantification, 1 μg of total RNA was reverse transcribed with random primers using PrimeScript^TM^ RT Master Mix (RR036A, TaKaRa, Japan). Then the expression levels of miRNAs and mRNA were quantified using a real-time PCR Detection System (Bio-Rad, United States). U6 and β-actin were used as the internal control for miRNAs and mRNA, respectively. All samples were conducted in triplicate on the same plate. The relative gene expression was calculated by the 2^–ΔΔCT^ method.

**TABLE 1:** The primers used in the qRT-PCR experiments.

Primer name	Sequence (5′–3′)
miR-221-3p RT-primer	GTCGTATCCAGTGCAGGGTCCGAGG TATTCGCACTGGATACGACGAAACCCA
miR-221-3p F	CGTCAAGCTACATTGTCTGCTG
miR-221-3p R	CAGTGCAGGGTCCGAGGTAT
U6 F	CTCGCTTCGGCAGCACA
U6 R	GCGTGTCATCCTTGCGC
IFN-α F	TCCTCCAACACCTCTTCGAC
IFN-α R	GGGCTGTAGGTGTGGTTCTG
IFN-β F	TCTACAGAGCCTTGCCTGCAT
IFN-β R	TGTCGGTGTCCAAAAGGATGT
SOCS5 F	TTCCTGCTCTACCAAAACCC
SOCS5 R	CTACTGTCCTGTTCGATACTGC
DTMUV-E F	AATGGCTGTGGCTTGTTTGG
DTMUV-E R	GGGCGTTATCACGAATCTA
β-Actin F	CCGGGCATCGCTGACA
β-Actin R	GGATTCATCATACTCCTGCTTGCT

*IFN, interferon; SOCS5, suppressor of cytokine signaling 5; DTMUV, duck Tembusu virus.*

### Western Blot

The transfected cells were washed gently using ice-cold phosphate-buffered saline (PBS) for three times and then lysed with radioimmunoprecipitation assay (RIPA) buffer (89900, Thermo Fisher, United States) containing 1% phenylmethylsulfonyl fluoride (PMSF) (36978, Thermo Fisher, United States) for 30 min on ice. After being boiled with 5× sodium dodecyl sulfate (SDS) loading buffer for 10 min, the lysates were separated by 12% SDS–polyacrylamide gel electrophoresis (PAGE) and transferred to polyvinylidene difluoride (PVDF) membranes (ISEQ00010, Millipore, United States). The membranes were blocked with 5% skim milk for 2 h at room temperature and then incubated with the rabbit anti-SOCS5 polyclonal antibody (GTX104722, GeneTex, United States) at 1:1,000 dilution or the mouse anti-E monoclonal antibody (prepared in our lab) at 1:2,000 dilution or anti-β-actin mouse monoclonal antibody (60008, Proteintech, China) at 1:2,000 dilution at 4°C overnight. Thereafter, the horseradish peroxidase (HRP)-conjugated goat anti-rabbit (A0208, Beyotime, China) and goat anti-mouse IgG (A0216, Beyotime, China) were used as the secondary antibody. Finally, the protein signals were detected using the enhanced chemiluminescence (ECL) reagent (1705060, Bio-Rad, United States).

### Dual-Luciferase Reporter Assay

The pmirGLO plasmid was observed in our laboratory. The wild-type 3′-UTR of SOCS5 was amplified by ordinary PCR, and the mutant SOCS5 3′-UTR was amplified *via* the overlap extension PCR method. The primers used are shown in Table 2. The PCR products were cloned into the pmirGLO plasmid between the *Sac*I and *Xba*I restriction enzyme sites. The recombinant plasmids were identified by restriction enzyme digestion and DNA sequencing. For the dual-luciferase reporter assay, pmirGLO-SOCS5-Wt or pmirGLO-SOCS5-Mut were co-transfected with miR-221-3p mimic or inhibitor into DEF using Lipofectamine 3000. Forty-eight hours after transfection, the firefly luciferase and Renilla luciferase activities were detected using Dual-Glo Luciferase Assay System (E2920, Promega, United States) according to the manufacturer’s protocol. The relative reporter activity was normalized by Renilla luciferase activity.

**TABLE 2:** The primers used in the dual-luciferase reporter assay.

Primer name	Sequence (5′–3′)
SOCS5-Wt F	TCCGAGCTCCTTCTCTATGGCAGTGACCT
SOCS5-Wt R	CTAGTCTAGATAAAATGCTCTATHCCTGCG
SOCS5-Mt F	CACGTAACCGACGTCAGTTAACTGAGTTTTAATAG
SOCS5-Mt R	GACGTCGGTTACGTGAATATATAAAAAC

### Virus Titer

DEF cells were seeded into 96-well plates and cultured overnight. The virus was serially diluted with DMEM from 10^1^- to 10^8^-fold containing 2% FBS, and the diluted virus with 100 μl was added to corresponding wells. Then the culture plates were incubated at 37°C in 5% CO_2_ incubator for 3–5 days until the appearance of cytopathic effects (CPEs). The CPE was observed under the microscope, and the TCID_50_ values were calculated using the Reed and Muench method (Reed and Muench, 1938).

### Statistical Analysis

Data are provided as means ± SEM, and *n* represents the number of independent experiments. All data were tested for significance using the unpaired Student’s *t*-test. Data were analyzed by Excel 2019 or GraphPad Prism Software 6.0, United States. *P*-value ≤0.05 was considered statistically significant.

## Results

### Duck Tembusu Virus Infection Upregulated miR-221-3p Expression

**FIGURE 1:**
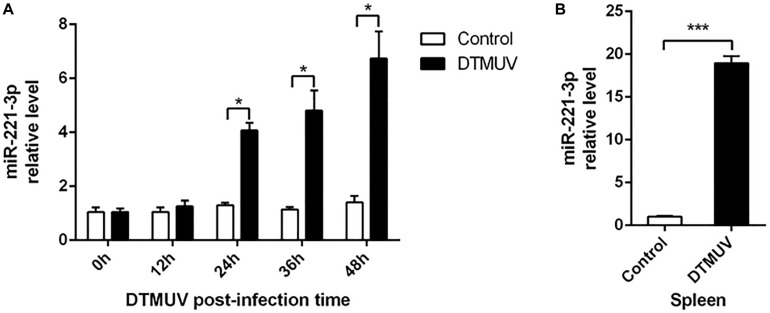
Duck Tembusu virus (DTMUV) infection upregulated miR-221-3p expression *in vitro* and *in vivo*. **(A)** The duck embryo fibroblast (DEF) cells were plated in 6-well plates and infected with the DTMUV at multiplicity of infection (MOI) = 1. Then, the cells were collected at the indicated times. RNAiso reagent was used to extract total RNA, and qRT-PCR analysis was used to determine miR-221-3p expression level. **(B)** The spleen tissue from DTMUV-infected ducklings and control spleen tissue were conserved in our laboratory. The miR-221-3p expression level from these tissues was detected by qRT-PCR analysis. U6 was used as an internal reference. All experiments were performed in triplicate (*n* = 3). *P < 0.05 and ***P < 0.001 indicate statistically significant difference using Student’s *t*-test.

Previously, we have described that the expression of miR-221-3p was changed in DEF in response to DTMUV infection (Cui et al., 2018). Here, we investigated the expression profiles of miR-221-3p in DTMUV-infected DEF cells. As shown in Figure 1A, the expression of miR-221-3p was significantly increased at 24, 36, and 48 h post infection in a time-dependent manner, when compared with that of control groups. Then, we further investigated whether DTMUV infection affected the miR-221-3p expression in ducks. The spleen tissues of uninfected and DTMUV-infected ducklings were collected at 3 days post infection in our laboratory. As shown in Figure 1B, the expression of miR-221-3p was increased about 20-folds than that of the control group. Taken together, these results indicated that the expression of miR-221-3p was upregulated upon DTMUV infection.

### Overexpression miR-221-3p Inhibited Interferon Production

**FIGURE 2:**
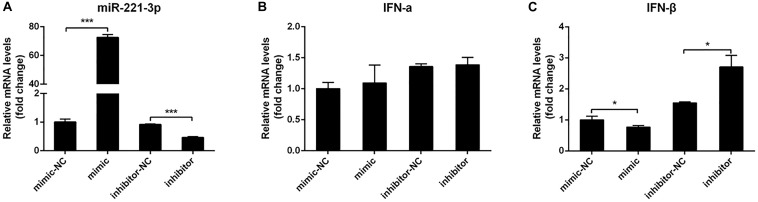
The effect of miR-221-3p on interferon (IFN) production. Duck embryo fibroblast (DEF) cells were transfected with 100 nM of mimic, 100 nM of mimic-NC, 200 nM of inhibitor, and 200 nM of inhibitor-NC for 24 h and then infected with duck Tembusu virus (DTMUV) at a multiplicity of infection (MOI) of 1. After 36 h of infection, total RNA was extracted using RNAiso reagent. The miR-221-3p **(A)**, IFN-α **(B)**, and IFN-β **(C)** expression levels were detected by qRT-PCR. U6 was used to normalize miRNA, and β-actin was used to normalize IFN mRNA. All experiments were performed in triplicate (*n* = 3). *P < 0.05 and ***P < 0.001 indicate statistically significant difference using Student’s *t*-test.

To date, many studies have demonstrated that miRNAs could regulate IFN production (Witwer et al., 2010; Forster et al., 2015). To explore the effect of miR-221-3p on type I IFN expression in DEF upon DTMUV infection, we transfected DEF with miR-221-3p mimic or inhibitor to measure the expression of type I IFN. Firstly, the miR-221-3p expression levels were detected in miR-221-3p mimic and inhibitor transfected DEF by qRT-PCR. As shown in Figure 2A, in mimic transfected cells, the expression of miR-221-3p was significantly increased about 70-folds than that of the mimic-NC transfected cells. We also confirmed that the expression of miR-221-3p was decreased in miR-221-3p inhibitor transfected cells compared with the inhibitor-NC transfected cells. Subsequently, we found that IFN-α expression was not affected in mimic and inhibitor transfected cells compared with their respective controls (Figure 2B). The expression of IFN-β was decreased in miR-221-3p mimic transfected cells, compared with the mimic-NC transfected cells. In contrast, the expression of IFN-β was increased in miR-221-3p inhibitor transfected cells, when compared with the inhibitor-NC transfected cells (Figure 2C). Therefore, overexpression miR-221-3p inhibited IFN-β production and inhibition of miR-221-3p promoted IFN-β production. These data indicated that miR-221-3p could negatively regulate the production of IFN-β.

### Overexpression miR-221-3p Promoted Duck Tembusu Virus Replication

**FIGURE 3:**
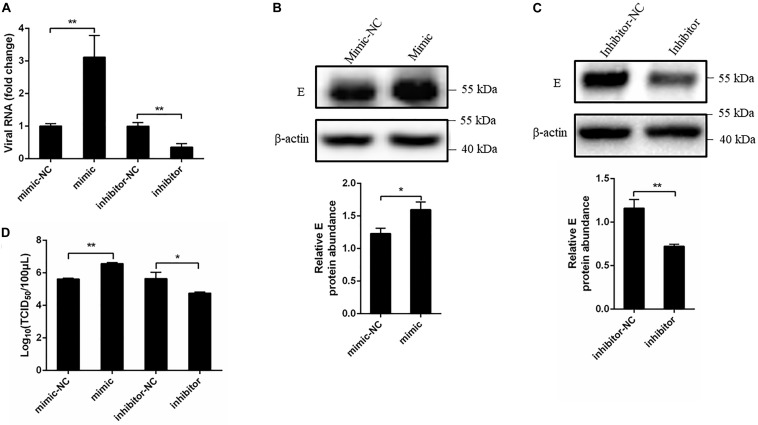
Overexpression of miR-221-3p facilitated duck Tembusu virus (DTMUV) replication. miR-221-3p mimic, inhibitor, and their respective controls were transfected into duck embryo fibroblast (DEF) cells. After 24 h of transfection, the cells were infected with DTMUV at a multiplicity of infection (MOI) of 1. **(A)** The cells were collected after 36 h of infection, and RNA was extracted using RNAiso reagent. The viral RNA level was determined by qRT-PCR. **(B,C)** The cells were lysed using radioimmunoprecipitation assay (RIPA) lysis buffer at 36 h post infection. The E protein and β-actin expression were detected by western blot. β-Actin expression was used as an internal control. The relative level of E protein expression was calculated as follows: the band density of E protein in each sample/that of β-actin in the same sample. **(D)** The cells were harvested at 60 h post infection for TCID_50_. All experiments were performed in triplicate (*n* = 3). *P < 0.05 and **P < 0.01 indicate statistically significant difference using Student’s *t*-test.

A growing body of evidence has demonstrated that miRNAs play a pivotal role in viral infection (Trobaugh and Klimstra, 2017). To explore the effect of miR-221-3p on DTMUV replication, we performed DEF transfected with miR-221-3p mimic or inhibitor and then followed it with DTMUV infection. Viral replication levels were determined through measuring viral copy number, E protein level, and viral titer. Results showed the viral copy number was significantly higher in the cells transfected with mimic than the cells transfected with mimic-NC (Figure 3A). Conversely, the viral copy number in inhibitor transfected cells was significantly lower than the inhibitor-NC transfected cells (Figure 3A). Subsequently, we tested the effects of miR-221-3p mimic and inhibitor on the DTMUV E protein expression level. As shown in Figures 3B,C, the E protein expression was increased in mimic-treated cells as compared with mimic-NC-treated cells, and the E protein expression was decreased in inhibitor-treated cells compared with inhibitor-NC cells. Furthermore, we tested whether overexpression or inhibition of miR-221-3p could affect viral titer by TCID_50_ assays. The results showed that viral titer in mimic transfected cells was enhanced than the mimic-NC group, and the viral titer was decreased by the inhibitor transfection (Figure 3D). Taken together, overexpression of miR-221-3p enhanced DTMUV replication and inhibition of miR-221-3p attenuated DTMUV replication. Thus, miR-221-3p could positively regulate DTMUV replication.

### Duck Tembusu Virus Infection Downregulated the Expression of Suppressor of Cytokine Signaling 5

**FIGURE 4:**
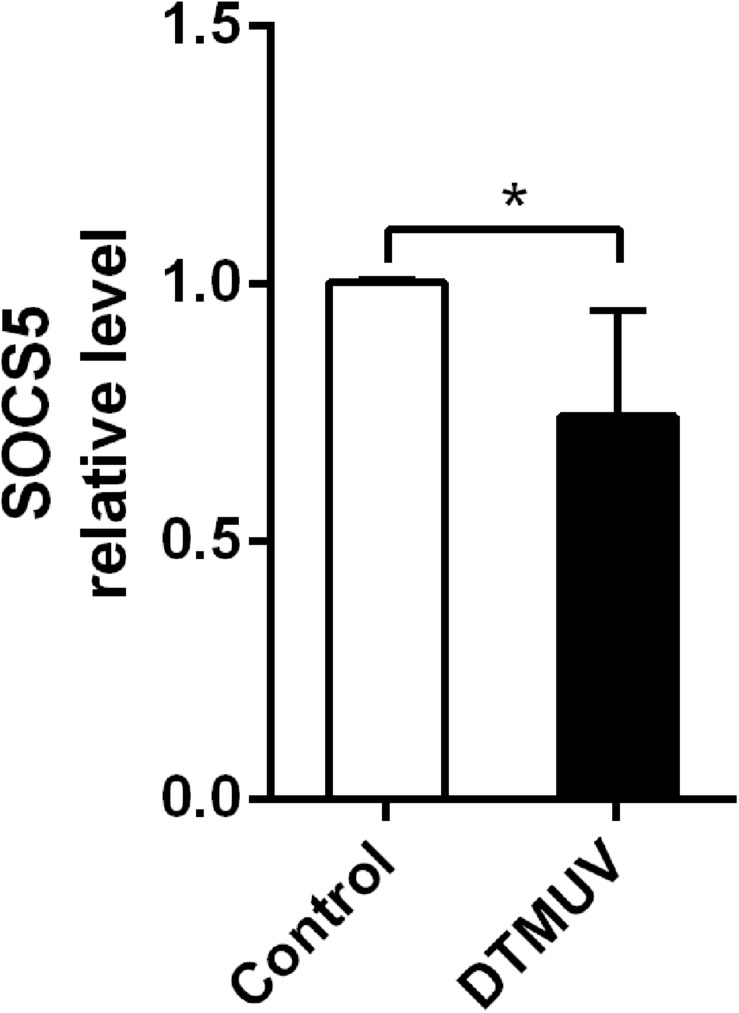
Duck Tembusu virus (DTMUV) infection downregulated suppressor of cytokine signaling 5 (SOCS5) expression in duck embryo fibroblast (DEF). The DEFs were plated in 6-well plates and infected with DTMUV at multiplicity of infection (MOI) = 1. Then, the cells were collected at 36 h post infection. The SOCS5 expression level was detected by qRT-PCR analysis. β-Actin was used as an internal reference. All experiments were performed in triplicate (*n* = 4). *P < 0.05 indicates statistically significant difference using Student’s *t*-test.

As we found DTMUV infection upregulated miR-221-3p expression, therefore, we considered that the upregulation of miR-221-3p may inhibit the expression of some host genes involved in DTMUV replication. Therefore, we predicted the potential target genes of miR-221-3p through bioinformatics analysis. SOCS5, a negative regulator of cytokines, attracted our attention. Then, we detected the expression of SOCS5 in DEF infected with DTMUV at 36 h *via* qRT-PCR analysis. As shown in Figure 4, the mRNA level of SOCS5 was reduced in DTMUV-infected DEF compared with the control group. Thus, DTMUV infection downregulated SOCS5 expression in DEF.

### Suppressor of Cytokine Signaling 5 Is a Target of miR-221-3p

**FIGURE 5:**
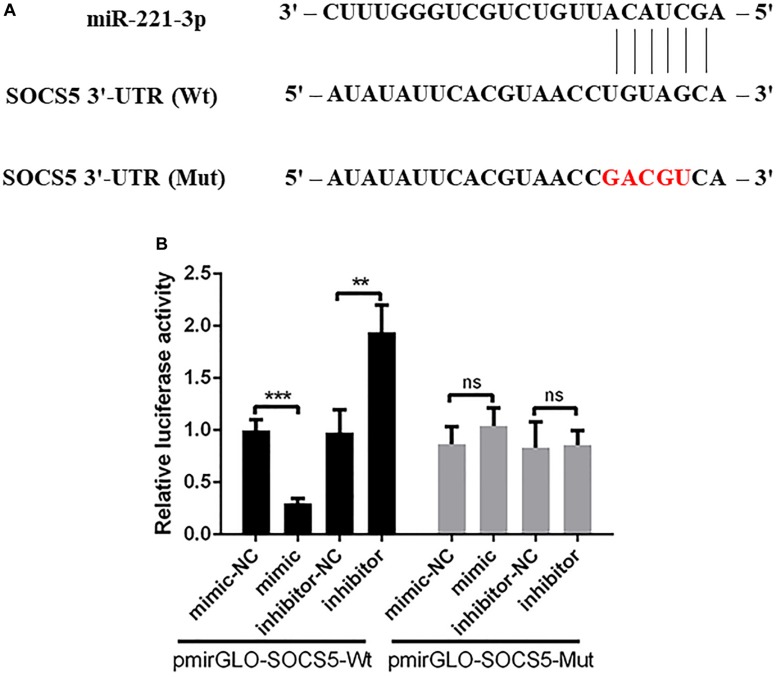
Suppressor of cytokine signaling 5 (SOCS5) is a potential target of miR-221-3p. **(A)** The schematic diagram showing the complementary sequences of the miR-221-3p binding site in the SOCS5 3′-UTR. The mutant binding sites are indicated in red letters. **(B)** The duck embryo fibroblast (DEF) cells were co-transfected with mimic, mimic-NC, inhibitor, and inhibitor-NC together with pmirGLO-SOCS5-Wt or pmirGLO-SOCS5-Mut. After 48 h of transfection, the cells were collected, and the luciferase activities were detected by dual-luciferase assays. All experiments were performed in triplicate (*n* = 3). ns indicates non-significance of difference. **P < 0.01 and ***P < 0.001 indicate statistically significant difference using Student’s *t*-test.

The bioinformatics analysis showed that a putative binding site was found in the 3′-UTR of SOCS5 (Figure 5A). Then we constructed the recombinant luciferase plasmids including SOCS5 3′-UTR wide-type or mutant-type using pmirGLO luciferase plasmid. The dual-luciferase reporter analysis showed that the relative luciferase activity was reduced in DEF treated with pmirGLO-SOCS5-Wt and miR-221-3p mimic compared with the mimic-NC group, and miR-221-3p inhibitor could significantly elevate the relative luciferase activity, whereas miR-221-3p mimic and inhibitor failed to significantly change the relative luciferase activity in DEF containing with the pmirGLO-SOCS5-Mut, compared with their respective control groups (Figure 5B). Therefore, these results indicated that SOCS5 is a target of miR-221-3p.

### miR-221-3p Reduced Suppressor of Cytokine Signaling 5 Expression at mRNA and Protein Levels

**FIGURE 6:**
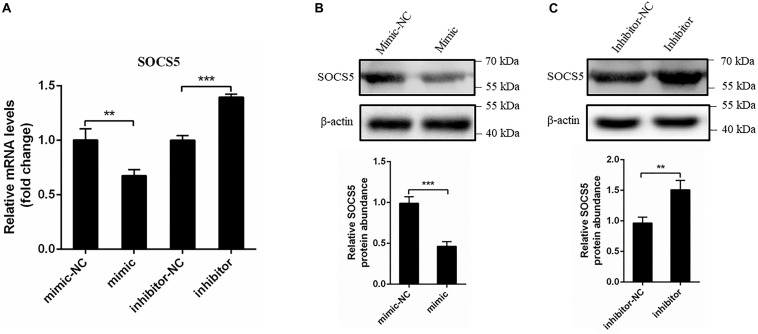
miR-221-3p downregulated the expression of suppressor of cytokine signaling 5 (SOCS5). Duck embryo fibroblast (DEF) cells were transfected with mimic, mimic-NC, inhibitor, and inhibitor-NC for 48 h. **(A)** The SOCS5 mRNA level was determined by qRT-PCR. **(B,C)** The SOCS5 protein level was detected by western blot. β-Actin expression was used as an internal control. The relative level of SOCS5 protein expression was calculated as follows: the band density of SOCS5 protein in each sample/that of β-actin in the same sample. All experiments were performed in triplicate (*n* = 3). **P < 0.01 and ***P < 0.001 indicate statistically significant difference using Student’s *t*-test.

We further determined whether miR-221-3p affects the mRNA and protein expression of SOCS5. The mimic or inhibitor was transfected into DEF for 48 h, then the SOCS5 mRNA level was detected *via* qRT-PCR, and the protein level was detected by western blot. As shown in Figure 6A, the SOCS5 mRNA expression was significantly decreased in mimic transfected cells compared with mimic-NC transfected cells, whereas the SOCS5 mRNA expression was improved in the inhibitor transfected cells. In accordance with the qRT-PCR results, western blot analysis showed that the SOCS5 protein expression was reduced by mimic transfection and the SOCS5 protein level was increased by inhibitor transfection (Figures 6B,C). Taken together, miR-221-3p could downregulate SOCS5 expression at mRNA and protein levels.

### Knockdown and Overexpression of Suppressor of Cytokine Signaling 5

**FIGURE 7:**
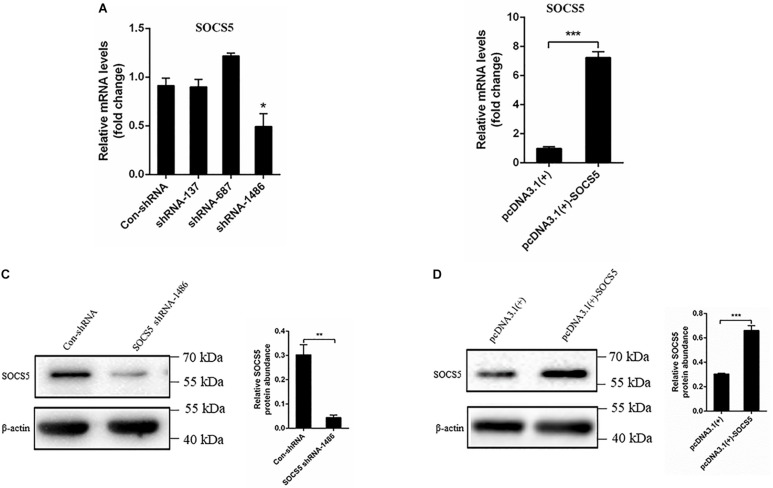
Knockdown and overexpression suppressor of cytokine signaling 5 (SOCS5). Duck embryo fibroblast (DEF) cells were transfected with con-shRNA, SOCS5 shRNAs, pcDNA3.1(+), and pcDNA3.1(+)-SOCS5 plasmids for 48 h. The SOCS5 mRNA expression level was determined by qRT-PCR. The differences of expression were determined using 2^–ΔΔCT^ method **(A,B)**. The SOCS5 protein expression level was measured by western blot. β-Actin expression was used as an internal control. The relative level of SOCS5 protein expression was calculated as follows: the band density of SOCS5 protein in each sample/that of β-actin in the same sample **(C,D)**. All experiments were performed in triplicate (*n* = 3). *P < 0.05, **P < 0.01 and ***P < 0.001 indicate statistically significant difference using Student’s *t*-test.

To examine the effect of the target SOCS5 gene during DTMUV infection, three shRNAs against SOCS5 were synthesized, and pcDNA3.1(+)-SOCS5 plasmid was constructed and then transfected into DEF. The SOCS5 expression was detected *via* qRT-PCR and western blot. The qRT-PCR results showed that SOCS5 mRNA expression was reduced after transfection with shRNA-1486, and SOCS5 mRNA expression was increased by pcDNA3.1(+)-SOCS5 plasmid transfection (Figures 7A,B). In agreement with qRT-PCR results, the western blot results also showed that SOCS5 protein expression was reduced by SOCS5 shRNA-1486 transfection and that SOCS5 protein expression was increased by pcDNA3.1(+)-SOCS5 plasmid transfection (Figures 7C,D). Therefore, these results proved that knockdown and overexpression of SOCS5 were successful.

### Knockdown of Suppressor of Cytokine Signaling 5 Enhanced Duck Tembusu Virus Replication

**FIGURE 8:**
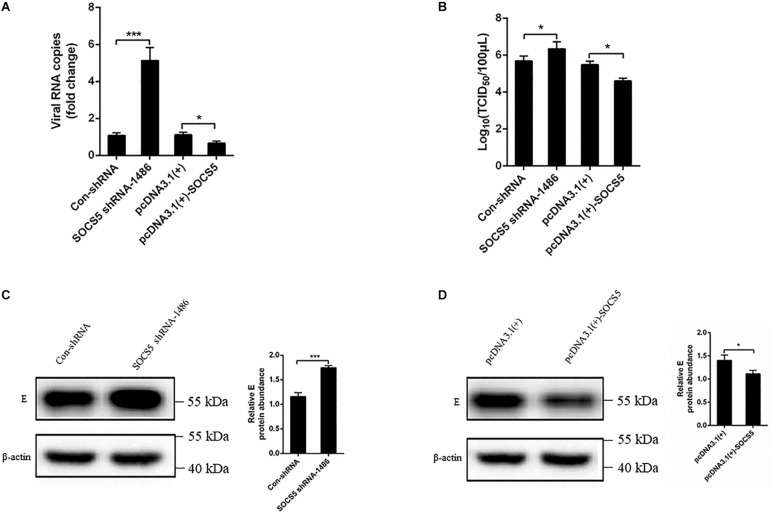
Suppressor of cytokine signaling 5 (SOCS5) inhibited Duck Tembusu virus (DTMUV) replication. Con-shRNA, SOCS5 shRNA-1486, pcDNA3.1(+), and pcDNA3.1(+)-SOCS5 plasmids were transfected into duck embryo fibroblast (DEF) for 24 h, followed by DTMUV infection at multiplicity of infection (MOI) = 1. After 36 h of infection, the viral RNA copies were determined by qRT-PCR **(A)**. After 60 h of infection, the cells were harvested for TCID_50_ **(B)**. After 36 h of infection, the viral E protein expression level was detected by western blot. β-Actin expression was used as an internal control. The relative level of E protein expression was calculated as follows: the band density of E protein in each sample/that of β-actin in the same sample **(C,D)**. All experiments were performed in triplicate (*n* = 3). *P < 0.05 and ***P < 0.001 indicate statistically significant difference using Student’s *t*-test.

We further detected the effect of SOCS5 on DTMUV replication. SOCS5 shRNA and pcDNA3.1(+)-SOCS5 were transfected into DEF and infected with DTMUV. The effect of SOCS5 on DTMUV replication was detected by qRT-PCR, TCID_50_, and western blot. The qRT-PCR and TCID_50_ results showed that the viral RNA copies and viral titer were significantly increased after knockdown of SOCS5 by transfecting SOCS5 shRNA-1486 (Figures 8A,B). In contrast, the viral RNA copies and viral titer were significantly decreased after overexpression of SOCS5 by transfecting pcDNA3.1(+)-SOCS5 (Figures 8A,B). The western blot results showed that DTMUV E protein expression was significantly increased after knockdown of SOCS5 and that DTMUV E protein expression was significantly decreased after overexpression of SOCS5 (Figures 8C,D). These results indicated that overexpression of SOCS5 could inhibit DTMUV replication and that knockdown of SOCS5 could enhance DTMUV replication.

## Discussion

**FIGURE 9:**
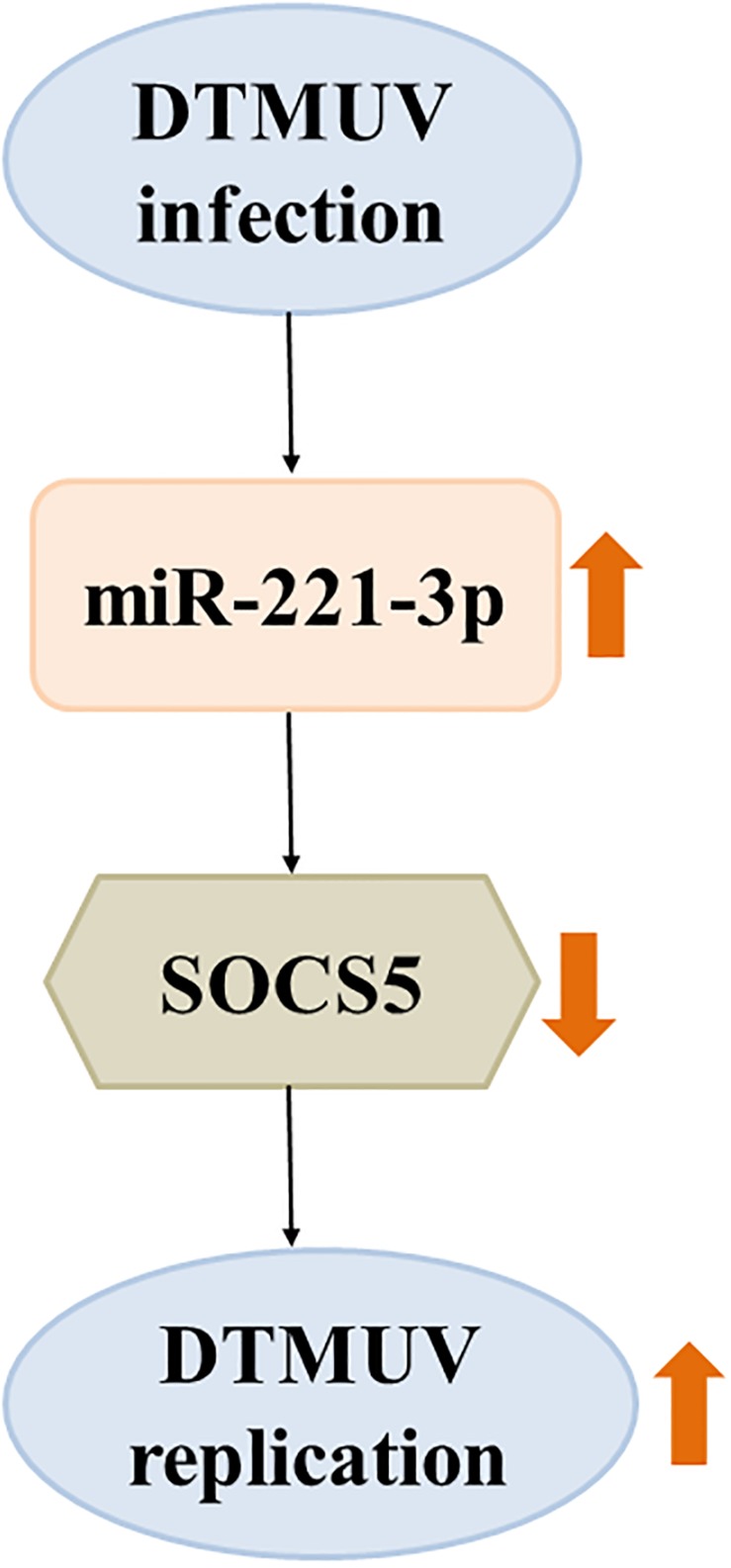
The schematic representation of the relationship between duck Tembusu virus (DTMUV) infection, miR-221-3p, and suppressor of cytokine signaling 5 (SOCS5) expression. DTMUV infection upregulated the expression of miR-221-3p. Overexpression of miR-221-3p inhibited the expression of its target, SOCS5. Suppression of SOCS5 promoted DTMUV replication. The thick orange arrows indicate the upregulation or downregulation of gene expression. The thick orange arrows indicate the positive or negative effect.

To date, there is increasing evidence that miRNAs play critical roles in viral replication and immune response. Our laboratory recently identified that 48 miRNAs were significantly differentially expressed in DEF upon DTMUV infection compared with uninfected DEF cells, and these dysregulated miRNAs may be involved in host–virus interaction (Cui et al., 2018). In this study, we further investigated the role of a significantly upregulated miR-221-3p in DTMUV replication. We indicated that overexpression of miR-221-3p decreased IFN-β production and promoted DTMUV replication. In detail, miR-221-3p could target SOCS5 functionally and inhibit its expression. Furthermore, we found that knockdown of the expression of SOCS5 promoted viral replication (Figure 9). To our knowledge, this is the first study that proved the regulatory relationship between cellular miR-221-3p and DTMUV replication. Therefore, our findings provide evidence that DTMUV increased the reproduction and proliferation of the virus itself by upregulating miR-221-3p, which represents a novel strategy adopted by DTMUV to evade host protective immune response that allows these organisms to survive.

It is well known that miRNAs could modulate viral replication *via* two distinct mechanisms. Recent studies indicated miRNAs could target viral genome to directly inhibit or promote viral replication. For example, miR-23, miR-378, and miR-505 inhibited PRRSV replication through binding to the viral genome (Zhang et al., 2014). miR-122 facilitated HCV replication *via* direct interaction with viral RNA (Norman and Peter, 2010). miR-281 enhanced DENV-2 replication through the interaction between the seed regions of miR-281 and the 5′-UTR of the DENV-2 sequence (Zhou et al., 2014). However, the binding sites of miR-221-3p in DTMUV UTR were not found in our bioinformatics analysis results. Of particular note, increasing studies showed that miRNAs indirectly modulated viral infection *via* targeting some host genes. For instance, upregulation of miR-146a enhanced JEV infection *via* targeting TRAF6, IRAK1, and IRAK2 (Sharma et al., 2015). Overexpression of miR-23b facilitated ALV-J replication *via* downregulating IRF1 (Li et al., 2015). In accordance with these results, we also indicated that overexpression of miR-221-3p reduced the IFN-β production and facilitated DTMUV replication. In a word, our results showed that the increased miR-221-3p during DTMUV infection negatively regulated IFN production and enhanced DTMUV replication. Therefore, we considered that miR-221-3p may target host genes that associated with the innate immune response to affect DTMUV replication. It is intriguing to further investigate the interaction mechanism of miR-221-3p in DTMUV infection.

Viral infection triggers the innate immune response (Satoshi and Shizuo, 2006; Koyama et al., 2008). Moreover, viral-induced immune response is also closely related to miRNAs-mediated post-transcriptional gene silencing mechanism (Xiao and Rajewsky, 2009; Chang Zheng et al., 2013; Islam et al., 2019; Rastogi and Singh, 2019). SOCS5, a negative regulator of cytokine and growth factor signaling, belonging to the SOCS family (Yoh Ichi et al., 2002; Edith et al., 2005; Nicholson et al., 2005; Kedzierski et al., 2017). SOCS proteins were involved in regulating immune response, tumorigenesis, and viral pathogenesis (Rottapel et al., 2002; Alexander and Hilton, 2004; Lisa Nowoslawski and Benveniste, 2011; Yakass et al., 2019). There are eight members of the SOCS family, including cytokine inducible SH2-containing protein (CISH) and SOCS1-7 (Delgado-Ortega et al., 2011; Duncan et al., 2017; Wang B. et al., 2019). Currently, a growing body of evidence has demonstrated that miRNAs level negatively correlated with SOCS protein expression (Peng et al., 2011; Jun et al., 2012; Yao et al., 2012; Wang X. et al., 2019). For instance, the expression of SOCS5 was inhibited by several miRNAs, such as miR-432, miR-301a, miR-130b, miR-124, miR-33-5p, miR-885-5p, and miR-132 (Shan et al., 2014; Sharma et al., 2016; Fu et al., 2017; Hazra et al., 2017; Diao et al., 2018; Su et al., 2018; Tsai et al., 2018). miR-432 inhibited JEV replication *via* regulating JAK-STAT signaling by targeting SOCS5, but another report indicated that miR-301a enhanced JEV replication through regulating EGFR signaling *via* targeting SOCS5 (Sharma et al., 2016; Hazra et al., 2017). The dysregulation of these miRNAs caused the change of SOCS5 expression, resulting in the modulation of JAK-STAT and EGFR signaling, thus regulating the immune response. Previous studies have demonstrated that SOCS5 is a negative regulator of host immune response by modulating JAK-STAT signaling to inhibit IFN response (Yoh Ichi et al., 2002; Hwang et al., 2007; Linossi et al., 2013). However, recent studies also indicated SOCS5 as a negative regulator of EGFR signaling to promote IRF1-mediated IFN response (Nicholson et al., 2005; Kedzierski et al., 2017). In our study, we indicated that SOCS5 was a direct target of miR-221-3p in DEF upon DTMUV infection, and knockdown of the expression of SOCS5 enhanced DTMUV replication. Taken together, our results showed that miR-221-3p could facilitate DTMUV replication *via* downregulating SOCS5. We speculated that DTMUV induced miR-221-3p expression *via* related molecular pathways during DTMUV infection, and then miR-221-3p targeted SOCS5 and inhibited its expression, which thus might enhance EGFR activity. The activation of EGFR may affect the expression and phosphorylation of IRF-7 of duck, resulting in inhibition of IFN-β (Chen et al., 2018). Inhibition of IFN-β weakens host innate immune response and facilitates the proliferation of the virus. The functional mechanism of miR-221-3p and SOCS5 in immune response and viral infection still requires further investigation.

## Conclusion

Taken together, we identified that DTMUV infection upregulated miR-221-3p expression, and we proved that the upregulation of miR-221-3p inhibited IFN production and enhanced DTMUV replication. Then, we further found that SOCS5 was a direct target of miR-221-3p and that miR-221-3p negatively regulated SOCS5 expression. Knockdown of SOCS5 could facilitate DTMUV replication. To our knowledge, this is the first study investigating the effect of miRNAs on DTMUV replication. The current study provides a new perspective for better understanding of host–virus interaction mechanisms and may provide a new strategy to design miRNA-based vaccines and therapies.

## Data Availability Statement

The datasets generated for this study are available on request to the corresponding author.

## Ethics Statement

The animal study was reviewed and approved by the Animal Ethics Committee of Sichuan Agricultural University (approval no. XF2016-17).

## Author Contributions

MC, SZ, AC, and RJ conceived and designed the experiments. MC and SC performed the experiments. MC, YP, XCZ, and ZH analyzed the data. MC and SZ wrote the manuscript. JH, MW, DZ, SLC, ML, XXZ, YW, QY, YL, LZ, YY, ZY, BJ, MR, BT, and LP revised the manuscript. All authors edited and approved the final manuscript.

## Conflict of Interest

The authors declare that the research was conducted in the absence of any commercial or financial relationships that could be construed as a potential conflict of interest.
